# Impacts of flood disasters in Nigeria: A critical evaluation of health implications and management

**DOI:** 10.4102/jamba.v11i1.557

**Published:** 2019-04-18

**Authors:** Caroline C. Olanrewaju, Munyaradzi Chitakira, Oludolapo A. Olanrewaju, Elretha Louw

**Affiliations:** 1 Department of Environmental Sciences, School of Ecological and Human Sustainability, University of South Africa, Johannesburg, South Africa; 2 Department of Industrial Engineering, Faculty of Engineering and the Built Environment, Durban University of Technology, Durban, South Africa; 3 Aurecon Centre, Cape Town, South Africa

## Abstract

Floods lead to tremendous losses of property, infrastructure, business and increased risk of diseases. Floods are also the most frequent natural disasters, affecting over 2.8 billion people in the world and causing over 200 000 deaths over the past three decades. The World Health Organization categorised the 2012 flood disaster in Nigeria as the worst flood to have hit the country in the past 50 years. This study reviews flood disasters in Nigeria and how they have been managed over the past two decades. The extensive review of the literature is complemented by data obtained from Ajegunle, a community in Ajeromi–Ifelodun Local Government Area. Because of its proximity to water bodies, its large population and its small land mass, the Ajegunle community is highly susceptible to floods and outbreaks of waterborne diseases. The study also discusses the institutionalisation and development of disaster management in Nigeria. Further, it critically evaluates the disaster management framework and other current disaster management policies as well as the effectiveness and functions of the disaster management focus areas and government response. The study takes a historic approach to flood disasters, linking disaster management to human health with a special focus on flood-related infectious diseases, isolating waterborne diseases as being predominant. Quantitative and qualitative data were collected to develop an understanding of how the people of Ajegunle are affected by flood disasters. This study reveals poorly managed health reforms and argues that in spite of government’s disaster management policies, there is an absence of organised and coordinated institutional structures to plan and respond to flood emergencies. It also revealed that diarrhoea outbreak was the predominant waterborne disease associated with flood disasters. Although Lagos State has been said to have the best flood preparedness plan in Nigeria, it has failed to reduce the yearly flood disasters and their impact on the health of the people. The article suggests a holistic approach by the government to get stakeholders, especially the health sector, more actively involved in disaster management planning.

**Keywords:**

disaster management; floods; waterborne diseases; Ajegunle; Lagos; Nigeria.

## Introduction

Floods are the most common naturally occurring hazard and are responsible for a greater number of fatalities globally (Doocy et al. 2013; Jonkman 2005). Floods are a result of excess water flowing on land that used to be dry (Djimesah, Okine & Mireku 2018). Among natural disasters, floods have been reported to be responsible for almost half of casualties (EM-DAT 2011). Floods are also the most frequent natural disasters, affecting over 2.8 billion people in the world and causing over 200 000 deaths over the past three decades (Hashizume 2013). Between 1995 and 2015, the lives of 2.3 billion people were affected, making floods accountable for 47% of all weather-related disasters globally (UNISDR 2015). Factors that cause flood events are complicated and interrelated (Halgamuge & Nirmalathas 2017). Floods are naturally caused by rise in temperature resulting in heavy downpours of rain, glacier melt and thermal expansion of the ocean, subsequently causing a rise in sea levels and inundation of coastal lands (Etuonovbe 2011). Climate change has been the major cause of these conditions globally. Floods are usually exacerbated by human activities such as construction of houses in areas that are prone to flooding (urbanisation) and deforestation (Byrant 1991).

Climate change threatens to block pathways out of poverty in developing countries, especially in Africa (Lemos & Tompkins 2008). An increase in disasters no matter the level will threaten development gains (ISDR 2008). Climate change is also expected to intensify disaster risk in the coming decade by causing more frequent and intense hazard events and increasing vulnerability of prone communities to the existing hazards (ISDR 2008). However, more focus now is on the Sustainable Development Goals, one of which is to mitigate climate change and its impacts by building resilience and limiting climate-related hazards and natural disasters (UN 2017). The impacts of floods in Nigeria are similar to what is experienced in other countries of the world such as Mali, Senegal, Burkina Faso and Niger (OCHA 2016), but the response may not be similar. Floods have led to tremendous losses of property, infrastructure, business and increased risk of diseases. For example, the Ogunpa flood, which occurred in Ibadan in 2011, resulted in a tremendous number of casualties. It was documented that about 25% of households in Ibadan lost their livelihoods, as their businesses were adversely affected (WHO 2012). Similar to Ogunpa were floods in the states along the rivers Niger and Benue in 2012 and 2017, Lagos in 2011, 2012 and 2017 as well as the Niger Delta regions in 2012.

When floods occur as natural incidents away from human populations, they have numerous benefits (Opperman, Galloway & Duvail 2013). However, when flooding occurs in areas of significant human development, especially in densely populated areas, a natural incident becomes catastrophic. Immediately after floods, there is poor hygiene and an increased risk of disease outbreaks, especially among displaced people (WHO n.d.). Potable water may be contaminated by pollutants from overflowing sanitation facilities, resulting in increased risk of waterborne diseases such as typhoid fever, cholera, leptospirosis and hepatitis A (WHO n.d.). Often poor people are more vulnerable and most affected (Yamin 2014). Health outcomes of floods are categorised into long- and short-term effects (Alderman, Turner & Tong 2012). Mortality rates tend to increase up to 50% globally within the first year after a major flood incident and psychological distress lingers for up to 2 years post-flood disaster with a prevalence of 8.6% to 53% (Alderman et al. 2012).

The study of the impact of floods on the health of the victims is very significant because it derails sustainable development. Several researches have been carried out on flood risk management and disasters in Nigeria. The weakness of flood risk management has been attributed to weak infrastructure, inadequate drainage network, absence of integrated flood risk management systems, weak institutions and poverty (Oladokun & Proverbs 2016). The World Health Organization assessed and proposed public health interventions after the 2012 flood disaster in Nigeria, which it categorised as the worst flood to have hit Nigeria in the past 50 years (WHO 2012). Ngutor (2015) also reviewed the 2012 flood disaster in Nigeria and its devastating effects that left many displaced and dead because of waterborne diseases and other risk factors. This study reviews past and present impacts of flood disasters on human health in the study area. It also reviews disaster management in Nigeria and critically analyses the government’s response using the National Disaster Management Framework (NDMF) as a guide to check government performance in the focus areas of the NDMF. It also reviews the different healthcare reforms in Nigeria over the study period and their participation in flood disaster management. This article seeks to contribute to the knowledge that will guide the development of responsible policies and structures targeted at combating floods and their ensuing health issues using Ajegunle, a community in Lagos State, as a case study.

### Floods and their health consequences

#### Lagos floods

Lagos, having a low-lying terrain, is the smallest state in Nigeria, with a total land area of 3577.28 km^2^, of which 22% or 787 km^2^ consists of lagoons and creeks (Oshodi 2012). According to the Lagos State Bureau of Statistics, Lagos is the smallest state in Nigeria but also the largest populated city in Nigeria, with a population of 10 203 million people (CIA World Factbook 2013), and it contains the largest urban area in Nigeria (Nigerian Finder 2013). Its southern border stretches 180 km along the coast of the Atlantic Ocean (Odunuga, Oyebande & Omojola 2012), with major water bodies in the area being the Lagos Lagoon and Ogun River; others traversing the area include Majidun and Aboyi Rivers. Lagos has a humid tropical climate as a result of its location along the coast and its proximity to the equator, with two distinct wet and dry seasons (Adelekan 2010). The rainy season occurs between the months of April and October. During the long rainy season, many parts of Lagos are susceptible to flooding and this is because of building of houses on flood plains, inadequate drainage of storm water, lack of maintenance of existing drainage systems, increased run-off because of uncontrolled expansions of impermeable surfaces and weak institutional capacity (Adelekan 2010). Lagos State ranks 15th in the world in terms of population exposed to coastal flooding (Sojobi, Balogun & Salami 2015).

The changes over the years of the pattern and intensity of rainstorms are also listed as factors influencing the risk of flooding (Adelekan 2010). A study performed in the period 1960–1980 showed that rainstorms were light and yielded less than 12.7 mL of rainfall (Ayoade & Akintola 1980). The analysis of rainstorms on Lagos Island carried out from 1971 to 2005 shows that in more recent times (1996–2005), heavier rainstorms have occurred in spite of a reduced number of rain days per annum. While fewer rain days were recorded during the 10-year period from 1996 to 2005, the mean annual rainfall was similar to the earlier period of 1971–1995. This is an indication that heavier rainstorms in the later periods were the cause of more flooding (Adelekan 2010). Land use changes and changes in hydrological fluxes of watersheds in urban settlements have been found to cause increasing flood hazards and risks in many parts of Lagos, most especially in slum communities (Adelekan 2010). Flooding in urban settlements in Lagos is compounded by inadequacy of the drainage network within the city. The problem has reached an alarming rate such that Adelekan (2010) proposed to the government some measures that can be put in place, which include but are not limited to enforcing urban planning laws, better ways of collecting wastes and construction of more drainage systems.

**FIGURE 1:**
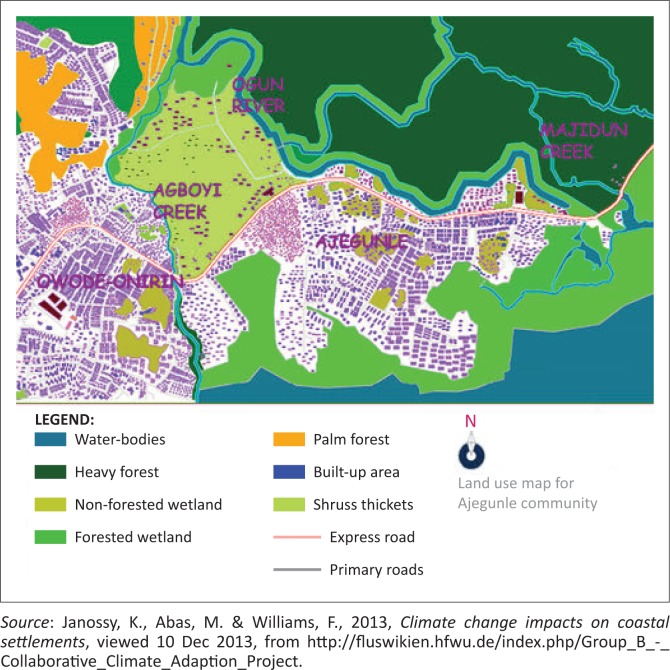
Land use map for the Ajegunle community, showing water bodies.

Like in any other sub-Saharan country plagued with overcrowding, poor sanitary conditions and lack of adequate medical and public health facilities, Ajegunle, popularly known as the ‘ghetto’ community, suffers from the consequences of floods. Flood disasters in Ajegunle are characterised by waterborne diseases, (CIA World Factbook 2013). Ajengule is a slum community that has existed for over 200 years and experienced water level rise over the years (Janossy, Abas & Williams 2013). It is a low-lying undulating flat land surrounded by rivers, creeks and a lagoon (see Figure 1). Its location on the coastal area makes it a high-risk area with respect to flooding in heavy rainfall seasons. Water released from the Oyan Dam after heavy rainfall contributes to this flooding. Whenever the Ogun River overflows its banks, water finds its way into Ajegunle and other adjoining communities. These floods last for several days, compromising the health of the people. Floods can be classified into different types according to the likelihood of their occurrence at a given period and geography. They include riverine floods, flash floods, coastal floods and urban floods (Means 2018). Because of its location, Ajegunle is susceptible to one or more of the above types of floods.

**FIGURE 2:**
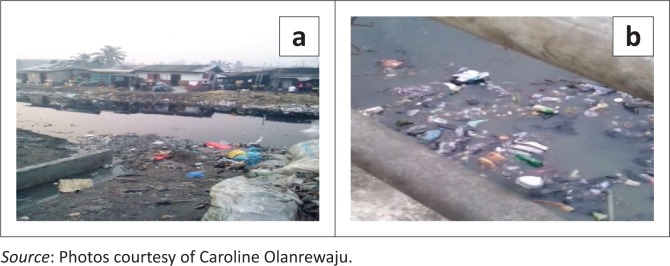
A section of Ajegunle with accumulation of stagnant water after a torrential downpour.

According to the Lagos State Bureau of Statistics, Ajeromi–Ifelodun Local Government Area (LGA) has the highest population density in Lagos State. A population projection carried out in 2007–2015 by the bureau using an annual growth rate of 3.2% projected a population density of 137 102, making it the most populous LGA in Lagos State (LG-Stat 2012). Ajegunle is a densely populated area with a density of 7941 pp/km^2^. It is in close proximity to two large ports in Nigeria, making it a commercial hub. The over-concentration of people in a relatively small space that is highly susceptible to floods is a recipe for outbreaks of waterborne diseases. Flooding increases the already compromised access to clean drinking water and sewerage, which has been adversely affected by haphazard development in the city. Despite the endowment of the water in the region, Ajegunle residents suffer from an acute shortage of water supply coupled with inadequate sewerage, causing human waste to be disposed of by means of rainwater through open ditches discharging onto the tidal flats. About 70% of the buildings are Brazilian type (high-rise story buildings with single-room apartments), which is characteristic of low income earners and poor communities in Nigeria; 13.1% are the traditional compound type and 9.2% are flats (Janossy et al. 2013). The majority of the roads are not tarred, characterised by poor drainage (see Figure 2), and some parts of the roadsides are used for illegal dumping. Whenever there is rainfall, most of the community is flooded, paving way for a massive outbreak of waterborne diseases.

Residents of Lagos live in apprehension of torrential rain. In 2010, scores of residents in Lagos and Ogun suffered a deluge occasioned by heavy rainfall aggravated by the release of water from the Oyan Dam into the Ogun River, causing the river to overflow its banks. As a result of this, 1000 residents were displaced in the wake of the disaster, which experts described as an epidemic threat to the affected communities (Okonkwo 2013). Ajegunle, an urban poor community situated along the low-lying coastal sites of Lagos, experiences annual flood attributed to rising sea levels. Floods associated with rising sea levels are related to climate change (Fitchett, Grant & Hoogendoorn 2016). Flooding in Lagos is linked to extreme rainfall, which is linked to climate change (Li, Guo & Yu 2013). Streams of river basins with smaller drainage area are known to respond quickly to climate change on shorter timescales ranging from hours to days to months (Chen et al. 2010). Located close to the Atlantic Ocean is a small deltaic drainage area; this makes the rivers and streams in Lagos State very sensitive to the extreme rainfall resulting from climate change and the impact of human activity (Sojobi et al. 2015). Also the frequency of storm surges may be exacerbated by sea level rise, especially at high tides and during the rainy season, characterised by heavy rainfall of long duration and intensity and increasing inadequacy of drainage systems (Adelekan 2010). Ajegunle has extremely poor environmental conditions including regular flooding of homes lasting for several hours, sweeping raw sewage and refuse into homes and causing pollution of potable water, leading to disease outbreaks such as cholera, dysentery, typhoid and paratyphoid fever and skin rashes (Adelekan 2010).

#### Health consequences

Waterborne diseases are the most commonly occurring infectious diseases (Isidore et al. 2012). Floods are the primary indicator of waterborne diseases coupled with poor sanitation around the recovery camps set up for the disaster victims. Waterborne diseases are infectious and endemic and present major primary health concerns following flooding. Flooding alters environmental equilibrium and affects the incidence and geographical range of climate-sensitive infectious diseases (Brown & Murray 2013). Intense precipitation mobilises pathogens in the environment and transports them into the aquatic environment, thereby increasing their microbial load on surface water (Peate, Wild & Nar 2013). Extreme torrential rain greater than 350 mm is a significant risk factor for enteroviruses and bacillary dysentery (Chen et al. 2012). The most common waterborne pathogens isolated after flooding are the *Vibrio* spp. (Cann et al. 2013). According to Brown and Murray (2013), excess rainfall causes waterborne disease outbreaks such as cholera, cryptosporidiosis, non-specific diarrhoea, rotavirus, typhoid and paratyphoid. They say this is a result of transportation of bacteria parasites and viruses into water systems. Onchocerciasis caused by black fly is endemic in Nigeria. This is because it is commonly found around quick-moving streams of the savannah and forest zones with 40 million people exposed to the disease, of which 22 million are infected and about 120 000 are estimated to be blind from the disease (Onwumere 2013). *The Tide* newspaper (Admin 2013) estimated that 868 000 children die annually from waterborne diseases.

Studies show that leptospirosis outbreaks occur after flooding because of lack of garbage management and collection following flooding where rubbish is left on the street, leading to an increased rodent population, especially rats (Lau et al. 2010). This is especially evident in overcrowded areas with poor sanitation, poor healthcare, poverty and abundance of rats and their animal reservoirs. While flooding may initially wash out vector population, they return when the waters recede, providing ideal breeding habitats for vectors (Brown & Murray 2013). During the September 2012 flooding that affected every part of Lagos, *Vanguard* online newspaper reported that the major challenge associated with the flood was waterborne diseases because of polluted drinking water and poor sanitation (Sessou 2012). It also recorded that people had to walk through the water to get to their homes, leading to skin diseases. According to reports, the floodwater came with lots of debris, faecal pathogens and pollutants. Individuals and organisations donated materially and financially to curb the menace. The Nigerian government put out 17.8 billion naira direct cash aids to affected states of the 2012 flood disaster (Federal Government of Nigeria [FGN] 2013). The European Union is one of the organisations that continue to help with water sanitation and hygiene in Nigeria through the United Nations International Children’s Emergency Fund (UNICEF) (Onwumere 2013).

## Methods

The research employed the quantitative and qualitative approaches. Ajegunle is comprised of 335 streets and 42 neighbourhoods (Asomba 2013). Five neighbourhoods, which include Aiyetoro, Okorogbo, Mba, Wowo and Alakoto were purposively selected for study. The areas were selected based on neighbourhoods that suffer the most flooding after heavy rainfall, have higher population density, have open market trading and high recorded waterborne diseases. Quantitative data were obtained from field-level questionnaires using closed-ended questions. The questionnaires had two sections. The first section comprised personal data with information on respondents’ age, sex, occupation, marital status, educational level, household size and annual income. The second section collected information about the impact of floods on the health of respondents, effects of waterborne diseases and government assistance. A total 280 questionnaires were distributed to 56 residents selected purposively based on availability and knowledge of floods from each of the five neighbourhoods. The qualitative data that depicted the perceptions held by the affected people in the community were obtained from key informant interviews with community heads, property owners, traders, taxi drivers, schoolteachers and nurses. In addition, on-the-spot assessments of the selected neighbourhoods were carried out by volunteers, who included a medical doctor, two teachers, a community pharmacist, three traders and two civil engineers. They assessed livelihood patterns, availability of pipe-borne water, infrastructure, proximity and conditions of healthcare facilities.

### Review of disaster management in Nigeria and the National Disaster Management Framework

#### Disaster management in Nigeria

Disaster management involves the coordination and integration of all activities necessary to build, sustain and improve the capabilities of communities to prepare for, protect against, respond to and recover from threats or actual natural or man-made disasters (NDMF 2010). Disaster management started in Nigeria in 1906 with the establishment of the Fire Brigade (now known as the Federal Fire Service), responsible for saving lives and property in addition to its primary function of firefighting and provision of humanitarian services during emergencies (Adelekan 2010). In 1972–1973, northern Nigeria suffered a devastating drought disaster with high socio-economic losses of lives and property worth millions of dollars. The impact of the disaster was so enormous that the government decided to create a response body to take care of disaster issues. This led to the creation of the National Emergency Relief Agency (NERA) by Decree 48 of 1976 (Shaba 2009). NERA was charged with the responsibility of collecting and distributing relief materials to disaster victims. However, based on the need for a holistic approach to disaster management, the name NERA was changed to National Emergency Management Agency (NEMA) to accommodate its expanded functions (Shaba 2009).

In March 1999, NEMA was established through *Act 12 of 1999* as amended by *Act 50 of 1999*. NEMA was given the responsibility of coordinating disaster management activities for the country (Nigeria-Government 2010) NEMA had roles and functions that were designed for a holistic approach to disaster management as stated in its mission statement. Their mission is:

to coordinate and facilitate disaster management efforts aimed at reducing the loss of lives and property and protect lives from hazard by the leading and support of disaster management stakeholders in a comprehensive risk based emergency management program of mitigation, preparedness response and recovery. (NEMA n.d.b)

The specific functions of NEMA include (1) disaster preparedness and mitigation activities; (2) notify, activate, mobilise and deploy staff as well as set up all necessary facilities for response; (3) evaluation and assessment of disaster damages; (4) management of funds for disaster; (5) inform and enlighten the public; (6) formulation of disaster management policies and guidelines in the country and (7) distribution of relief materials to disaster victims by liaising with State Emergency Management Committees, non-governmental organisations (NGOs), regional and international bodies (NEMA 2004a; Shaba 2009). The organisational structure of NEMA is made up of five main departments and three units. These comprise the following: search and rescue, relief and rehabilitation, training, finance and administration, a public relations unit, legal unit and audit unit. The objectives of NEMA are achieved by collaborating with state government, local government, voluntary organisations, international agencies and 57 disaster response units scattered all over the country (Ndiribe 2010; NEMA n.d.b).

In August 2006, zonal offices of NEMA were opened in the six geopolitical zones of the country to take disaster management to the community level. The functional State Emergency Management Agency (SEMA) backed by law with full operational capability was established in states to enhance proximity to the communities for the purpose of communication and coordination (NEMA 2004b).

Local Government Emergency Management Committees were established in response to calls from communities with strong facts that disaster strikes are felt mostly in communities (Nigeria-government 2010). The synergy of the three jurisdictional organisations (federal, state and local government) centres on the principle of shared responsibility and the leverage to ensure proper integration and collaboration among stakeholders and reduce the likelihood and severity of disasters (NDMF 2010). The Grassroots Emergency Volunteer Corps was carved out to give communities the capacity to respond to threats themselves because these communities are at the forefront of the disasters, referred to as ‘disaster fronts’ (Shaba 2009). The involvement of different stakeholders and actors in disaster management made it essential to have a mechanism to collaborate and coordinate activities. This mechanism is provided by the NDMF, which serves as a regulatory guideline for effective and efficient disaster management in Nigeria.

Other current documents that complement the *NEMA Act* include the Search and Rescue and Epidemic Evacuation Plan (NEMA 2011b), the National Contingency Plan of Nigeria (NEMA & UNICEF 2011) and Lake Nyos Disaster Response Plan (NEMA 2011a). The Search and Rescue and Epidemic Evacuation Plan is responsible for action plans in nine disaster scenarios, namely floods, fires, rail accidents, collapsed buildings, maritime-related disasters, oil spill disasters, aviation disasters, epidemics and road traffic accidents (Atala 2011). The Search and Rescue and Epidemic Evacuation Plan effectively coordinates at the scene of the disaster. The availability and promptness of personnel and materials at the scene leaves much to be desired. The National Contingency Plan of Nigeria focuses on hazards having the highest probability of occurrence and severity, such as floods, droughts, epidemics and communal conflicts. The contingency plan defines the modus operandi for the engagement of international assistance when it is required. The plan was tested and limited to a population of 10 000 and so has suffered a lot of setbacks, as it is not pragmatic and workable in a very high population (NEMA & UNICEF 2011). During the 2012 flood disaster, the contingency plan could not be implemented, as over 5 million people were affected. The limitation to the existing plan calls for a better plan that can accommodate the population of a state in the country to say the least.

#### National Disaster Management Framework of Nigeria

There is a need for all stakeholders and various actors in disaster management to exist in a coordinated and collaborative mechanism. The NDMF provides this mechanism, which led to its establishment in 2010 (Okoli 2014). NDMF defines coordinating structures that are measurable, adaptable and flexible and aligns key roles and responsibilities of disaster management stakeholders across the nation, describing specific authorities and best practices for managing disasters and explaining a paradigm shift in disaster management beyond mere response and recovery, offering a holistic approach to disaster management (NDMF 2010). The NDMF serves as a legal instrument to address the need for consistency among multi-stakeholders. It provides coherency, transparency and inclusive policy for disaster management in Nigeria. The framework was written so that government officials, civil society organisations, private sector, emergency management practitioners and community leaders can understand the concept and operating guidelines of disaster management in the country.

The NDMF focuses on eight sections made up of seven focus areas and sufficiency criteria. These include institutional capacity, Coordination, Disaster Risk Assessment, Disaster Risk Reduction, Disaster Prevention, Preparedness and Mitigation, Disaster Response, Disaster Recovery, Facilitators and Enablers (Nigeria-government 2010).

While the efforts of the Nigerian government are appreciated in the NDMF plan laid out, the response of NEMA falls short of effective disaster management requirements. The focus is mainly on drawing up response strategies rather than prevention and reduction because of financial, equipment, accommodation and mobility challenges (Aladegbola & Akinlade 2012). Inadequate funding is said to be a major cause for the failure of the NDMF. Proper, adequate and prompt government funding is very essential to determine the efficacy and effectiveness of the NDMF. Adequate material resources to carry out allocated tasks are lacking (Aladegbola & Akinlade 2012). Equipment and technology to predict, detect and mitigate disasters and the building of human capacity are needed (Adefisoye 2015). The Nigerian government’s failure to effectively manage disasters can be attributed to poor planning, response and management, taking into account the yearly flood disasters experienced in Lagos (Aladegbola & Akinlade 2012) and other coastal regions in Nigeria. Annual flooding is experienced in Lagos usually from the month of July to October with increasing frequency and severity of impact (Nkwunonwo, Whitworth & Baily 2016).

Responses to emergency calls have been very poor. Notable ones are the flood disasters in 2011 and 2012, which point to poor coordination of activities (Adefisoye 2015). Flood management in Lagos is being queried in terms of effectiveness and management policies (Adelekan 2016), lack of early warning and evaluation systems (Nkwunonwo et al. 2016) and lack of data and poor data scale. In the 774 LGAs in Nigeria, response initiatives are worst and emergency services are dysfunctional because state governors failed to ensure that democratic structures are institutionalised at the grassroots level of the 36 states and 774 local councils (Onwabiko 2012). Most of the states with SEMA have not assumed optimal operation since their existence (Adefisoye 2015). According to Adefisoye (2015), there is a lack of full backing by the law added to the non-conformity and non-compliance of its provision at the LGA. Research revealed that the functions of NDMF are not carried out especially in the SEMA and Local Government Emergency Management Agency (LEMA) because NEMA is not empowered by law to punish them (Adefisoye 2015).

Lately, the NDMF has incorporated operations such as improvement of general flood awareness through the National Orientation Agency, flood warning via Nigeria Hydrological Services Agency and integration of local, state and government emergency management agencies (Nkwunonwo et al. 2016). Despite all this, the NDMF is criticised as weak as the roles of this institution are not clearly defined (Adelekan 2016; Nkwunonwo et al. 2014).

### Review of healthcare delivery in Nigeria

Healthcare service delivery is a vital factor for the sustainable development of any nation (Briggs-Iti 2012). Nigeria’s healthcare has suffered various downfalls according to the Health Reform Foundation of Nigeria (HERFON n.d). Healthcare is greatly underserved in Nigeria. Health centres, personnel and medical equipment are inadequate, especially in rural areas. This was evident during one of the visits to Ajegunle. While reforms like the Nigeria Health Insurance Scheme (NHIS) put forward by the Nigerian government are operational at the national level, they are yet to be implemented at the state government level (Monye 2006) and by extrapolation the local government level. According to the 2009 communique of the Nigerian National Health Conference (NNHC 2009), healthcare systems remain weak as presented by lack of coordination, inadequate and decaying infrastructure, inequality in resource distribution, fragmentation of services and deplorable quality of care. A lack of clarity of roles and responsibilities among different levels of government compounds the situation.

Healthcare provision in the country remains a primary function of the three tiers of government – federal, state and local (Adeyemo 2005). Primary healthcare system is managed by the existing 774 LGAs in Nigeria with support from their respective ministries of health in the states and private medical practitioners (Omoruan, Bamidele & Phillip 2009). There are also sublevels of primary healthcare at the village, district and LGA levels. The Ministry of Health at the state level manages secondary healthcare system. Patients from primary healthcare are referred to secondary healthcare. The teaching hospitals and specialist hospitals provide tertiary healthcare. At the tertiary level, the government also works with voluntary organisations, NGOs and private practitioners (Adeyemo 2005).

The Nigerian healthcare system has weathered several infectious disease outbreaks and chemical poisoning occurrences over decades (HERFON n.d). Several studies have reviewed the Nigerian healthcare system and offered possible recommendations to improve the state of healthcare in the country. Several healthcare reforms have been launched in Nigeria by the federal government to revitalise the worsening state of health over the years (Awosika 2005) – for example, the 10-year development plan from 1946 to 1956, the Primary Healthcare Plan of 1987 and the NHIS, established in 2005 by Decree 35 of 1999. The primary healthcare plan made little impact on the healthcare sector as it continued to suffer major infrastructural and personnel inadequacies as well as poor public health management (Welcome 2011). According to the review by Welcome (2011), the NHIS has hardly attained any success, as there is continued limited healthcare delivery, no equitability and lack of access by the majority of Nigerians as reflected by high infant mortality, poor maternal care, low life expectancy, periodic outbreaks of the same diseases and inadequate control of the various outbreaks.

The inadequacy of the healthcare delivery system in Nigeria could be directly attributed to the following demographics of the Nigerian population. About 55% of the Nigerian population lives in rural areas and approximately 45% lives in the urban areas (Omoruan et al. 2009). About 70% of the healthcare is provided by the private sector and 30% by the government (Omoruan et al. 2009). Over 50% of the population live below the poverty line of less than $1.90 a day and cannot afford the high cost of health services (Omoruan et al. 2009). No adequate and functional surveillance systems have been developed, thus there is no tracking system to monitor the outbreak of communicable diseases (Welcome 2011).

Disease outbreaks occur long after flood disasters, especially in densely populated areas. Healthcare is typically withdrawn a few weeks after the disaster – not long enough for the impact of the disaster to be felt on the health of the people. Several factors exacerbate proper management of the health impact of floods, such as the media and anxiety of healthcare practitioners, which lead to panic, confusion and misplaced public health activities (Kouadio et al. 2012).

### Ethical considerations

The project complied with the research ethics requirement granted by the University of the Free State.

## Results and discussion

The results of the questionnaires showed that 47.1% suffer from diarrheal outbreak (cholera and dysentery) as supported by the findings of Adelekan (2010) and Brown and Murray (2013). Cann et al. (2013) also found that the most common waterborne pathogen isolated after flooding was the *Vibrio* spp., which causes cholera (watery diarrhoea).

The high prevalence of diarrheal outbreak was attributed to contaminated drinking water from destroyed sanitary infrastructure and sewage systems, as documented by Sessou (2012) in the review of the 2012 flood disaster. Moreover, 21.7% suffered from typhoid fever, which is as a result of contamination of food or water by faecal matter from broken sewage systems, found to be a major problem during flooding in the community. A further 17.5% suffered from malaria fever. These cases occurred because of collection of stagnant water in potholes of roads and blocked drainages, rivers and lagoon having waste and garbage in them that prevented their free flow. In addition, 4.3% suffered from skin rashes because of contact of the skin with polluted water for prolonged and repeated times. Likewise, 3.6% and 1.1% were found to suffer from hepatitis A and E, respectively. Leptospirosis sufferers were 1.8%, resulting from rodents such as rats urinating in the water thus polluting it, as noted by Lau et al. (2010). Schistosomiasis accounted for 0.4% of the cases and other water-related infections accounted for 2.5% (see Table 1).

**TABLE 1:** Result of questionnaire showing major waterborne disease outbreak experienced in Ajegunle.

Waterborne disease	Frequency	Percentage (%)
Diarrheal outbreak (cholera and dysentery)	132	47.1
Leptospirosis	5	1.8
Hepatitis A	10	3.6
Hepatitis E	3	1.1
Malaria fever	49	17.5
Skin rashes	12	4.3
Typhoid fever	61	21.7
Schistosomiasis	1	0.4
Others	7	2.5

**Total**	**280**	**100.0**

During the course of the interview, much information was revealed. The impacts of floods on the well-being of the people were devastating and derailed the community’s economic development. This survey confirmed that the effects of the 2012 flood disaster caused human deaths from disease outbreaks such as cholera and dysentery, especially in children below the age of 5 years and the elderly, primarily because of pollution of water by sewage and garbage, as also documented by Sessou (2012) and the WHO (n.d.). The impact of the disaster was made worse because healthcare facilities were not accessible because of flooded roads and because healthcare personnel did not have ready access to the affected areas. There was migration of new people into the community every year, thereby increasing the population of the community without matching improvement of services by the government. Help and donations coming from governmental organisations and NGOs, as noted by Onwumere (2013) and FGN (2013), is supposed to be delivered immediately after the flood disasters, but they are yet to be felt in many communities in Ajegunle. The inhabitants of Ajegunle were found to be predominantly traders and menial workers, and because floods keep traders away from work this affects their productivity. The WHO (2012) made similar observations in the case of the Ogunpa flood in Ibadan in 2011. The community’s vulnerability to flood disasters can be explained by poor infrastructure such as roads, poor waste disposal management, overpopulation, poor healthcare delivery and lack of health facilities. The lack of early warnings and knowledge about future flood disasters among the community members as well as poor government response to flood disasters especially during the response phase help to explain the magnitude of the resultant effect. Adefisoye (2015) observed in addition that poor coordination of relief and rescue activities worsen the situation. The majority of community members do not have coping strategies but take the flood disaster as an act of God that is beyond their control, and all they do thereafter is rebuild their houses and businesses on the same spot.

## Conclusion

The study revealed that the community under focus suffered from development setbacks as a result of persistent floods, thus affecting the livelihoods and general well-being of the people. Several households lost children to diarrheal outbreaks, malaria and typhoid fever resulting from stagnant and polluted drinking water.

It was also observed that after the floods, garbage was littered all over the area, providing habitat for rats, snakes, scorpions and other harmful insects. Rivers and the lagoon were crowded with debris, preventing them from flowing. Markets were covered with mud and garbage, and roads developed several potholes, storing dirty water that provided breeding grounds for mosquitos.

Even though the Nigerian government has drawn up several intervention strategies, it has been unsatisfactory in ameliorating the sufferings of flood victims. To a larger extent, the government response and policies have not been efficient and the recovery process has been slow. Several factors have been found to be responsible for these shortcomings, which explain the inability of the people to respond and cope with flood disasters. These factors include lack of restructuring and rebuilding functionality towards mitigating risks associated with floods. In the absence of pre-surveillance data, which is a big issue in Nigeria and other developing countries, risk assessment becomes very difficult to address. Urban poor communities such as Ajegunle feel most of the impacts of floods and the consequences on health. In spite of the recommendations by government and NGOs as well as health reforms, waterborne diseases continue to be a huge setback in the health sector. However, with knowledge of the prevalent waterborne diseases in Ajegunle and information from ongoing research by scholars, it is hoped that floods and the associated waterborne diseases will be reduced significantly.

## Recommendations

Based on the review of the existing framework, four phases of managing disaster (Djimesah et al. 2018) and visual inspection of the affected area, the following is recommended:

- With a population of over 10 million, Lagos State needs a contingency plan in the form of four phases of managing disaster – prevention; preparedness; response; and recovery – that can cater for a very high population. More focus should be placed on the disaster preparedness of the urban poor communities. Market men and women, schoolchildren, the illiterate and literate alike should be continually trained on flood disaster risks to build their listawareness.
- Responsible authorities should ensure that donations from volunteer groups reach the desired population. This can be achieved by giving these groups direct access to the victims and not passing through government storehouses where they could be redirected for other uses. Resource distribution by governmental organisations should be done according to the needs of the affected population and not on the same scale level as affected areas.
- Based on visual inspection during the site visitation, temporary clinics should be set up at the scene of rehabilitation camps and should contain drugs and amenities needed by victims especially in urban poor communities, as medical services are otherwise extremely expensive. The government should accord free medical care to flood victims, especially psychological care for post-traumatic incidences.
- Flood control strategies should be regularly updated by the different tiers of government. There should also be regular inspection on adherence to land policies by the Ministry of Lands and Environment, which will put land owners in check on encroachment into wetlands and other restricted areas.
- Institutional frameworks at the tiers of government, especially the local government, should be strengthened. Medical reforms should be implemented at the state and LGA levels and coordination between the tiers of government strengthened. There should also be improvement of waste management plans by the Federal Ministry of Environment.
- Government healthcare establishments should be regularly renovated and the equipment updated to match the private sector. In this way, public health centres can competitively cater for the urban poor communities, providing them with rapid implementation of control measures in disease outbreaks following flood disasters.

## References

[CIT0001] Adefisoye T., 2015, ‘An assessment of Nigeria’s institutional capacity in disaster management’, *Scientific Research Journal (SCIRJ)* 3, 37–48.

[CIT0002] Adelekan I., 2010, ‘Vulnerability of poor urban coastal communities to flooding in Lagos, Nigeria’, *Environment and Urbanization* 22, 433–450. 10.1177/0956247810380141

[CIT0003] Adelekan I.O., 2016, ‘Flood risk management in the coastal city of Lagos Nigeria’, *Journal of the Flood Risk Management* 9(3), 255–264.

[CIT0004] Adeyemo D.O., 2005, ‘Local government and healthcare delivery in Nigeria: A case study’, *Journal of Human Ecology* 18, 149–160. 10.1080/09709274.2005.11905822

[CIT0005] Admin, 2013, ‘Water-borne diseases claim 868,000 children’, *The Tide Newspaper,* 11 March, viewed 31 January 2016 from http://www.thetidenewsonline.com/2013/03/22/water-borne-diseases-claim-annually-868000-children/.

[CIT0006] Aladegbola I.A. & Akinlade M.T., 2012, ‘Emergency management: A challenge to public administration in Nigeria’, *International Journal of Economic Development Research and Investment* 3, 82–90.

[CIT0007] Alderman K., Turner L.R. & Tong S., 2012, ‘Foods and human health: A systematic review’, *Environment International* 47, 37–47. 10.1016/j.envint.2012.06.003 22750033

[CIT0008] Asomba I., 2013, *Horrible link road: Ajegunle on the verge of isolation,* viewed 12 August 2013, from http://www.vanguardngr.com/2013/05/horrible-link-road-ajegunle-on-verge-of-isolation/.

[CIT0009] Atala T., 2011, *An appraisal of the legal framework of the National Emergency Management Agency in the management of Internally Displaced Persons in Nigeria*, Ahmadu Bello University (Abu) Zaria, Nigeria

[CIT0010] Awosika L., 2005, ‘Health insurance and managed care in Nigeria’, *Annals of Ibadan Postgraduate Medicine* 3, 40–46.

[CIT0011] Ayoade J.O. & Akintola F.O., 1980, ‘Public perception of flood hazard in two Nigerian cities’, *Environment International* 4, 277–280. 10.1016/0160-4120(80)90079-3

[CIT0012] Briggs-iti I., 2012, ‘Natural Disasters and the role of environmental health practitioners’, *41st Annual National Conference/Scientific workshop for Environmental Health Officers Association of Nigeria (EHOAN)*, Yenagoa, Bayelsa State, Nigeria, March 2008.

[CIT0013] Brown L. & Murray V., 2013, ‘Examining the relationship between infectious diseases and flooding in Europe: A systemic literature review and summary of possible health interventions’, *Disaster Health* 1, 1–11. 10.4161/dish.25216 28228994 PMC5314884

[CIT0014] Byrant E.A., 1991, *Natural hazards*, Cambridge University Press, New York.

[CIT0015] Cann K.F., Thomas D.R., Salman R.L., Wyn-Jones A.P. & Kay D., 2013, ‘Systemic review: Extreme water-related weather events and water-borne diseases’, *Epidemiol Infections* 141, 671–686. 10.1017/S0950268812001653 PMC3594835 22877498

[CIT0016] Chen M.J., Lin C.Y., Wu Y.T., Lung S.C. & Su H.T., 2012, *Effect of extreme precipitation to the distribution of infectious diseases in Taiwan, 1994–2008 PLoS One* 7(6), e34651 10.1371/journal.pone.0034651 PMC3380951 22737206

[CIT0017] Chen Y.D., Zhang Q., Xu C.Y., Lu X. & Zhang S., 2010, ‘Multiscale streamflow variations of the pearl river basin and possible implications for the water resource management within the pearl river delta, China’, *Quaternary International* 226, 44–53. 10.1016/j.quaint.2009.08.014

[CIT0018] CIA World Factbook, 2013, *Nigeria’s major infectious disease: Nigeria,* viewed 15 December 2013, from https://www.cia.gov/library/publications/the-world-factbook/fields/2193.html.

[CIT0019] Djimesah I.E., Okine A.N.D. & Mireku K.K., 2018, ‘Influential factors in creating warning systems towards flood disaster management in Ghana: An analysis of 2007 Northern flood’, *International Journal of Disaster Risk Reduction* 28, 318–326. 10.1016/j.ijdrr.2018.03.012

[CIT0020] Doocy S., Daniels A., Murray S. & Kirsch T., 2013, ‘The human impacts of floods: A historical review of events 1980–2009 and systematic literature review’, *PLoS Currents Disasters*. Edition 1 page 1–29. 10.1371/currents.dis.f4deb457904936b07c09daa98ee8171a PMC3644291 23857425

[CIT0021] EM-DAT, 2011, *The OFDA/CRED International Disaster Database*, viewed 07 August 2017, from www.emdat.be/database.

[CIT0022] Etuonovbe A.K., 2011, *The devastating effect of flood in Nigeria, Hydrography and environment TS06J*, Epworth, Marrakech, Zimbabwe.

[CIT0023] Federal Government of Nigeria (FGN), 2013, *Nigeria post disaster needs assessment 2012 floods*, FGN, Abuja.

[CIT0024] Fitchett J.M., Grant B. & Hoogendoorn G., 2016, ‘Climate change threats to two low-lying South African coastal towns: Risks and perceptions’, *South African Journal of Science* 112, 1–9. 10.17159/sajs.2016/20150262

[CIT0025] Halgamuge M.N. & Nirmalathas A., 2017, ‘Analysis of large flood events: Based on flood data during 1985–2016 in Australia and India’, *International Journal of Disaster Risk Reduction* 24.

[CIT0026] Hashizume M., 2013, ‘Precipitation and flood hazards: Health effects, risk and impacts’, *Climate Vulnerability* 1, 115–121.

[CIT0027] HERFON n.d., *Health reform of Nigeria*, viewed 04 April 2014, from www.herfon.org.ng/

[CIT0028] ISDR, 2008, *Disaster risk reduction strategies and risk management practices: Critical elements for adaptation to climate change*, viewed 20 December 2013, from www.unisdr.org/…/risk-reduction/climate-change/…/IASC-ISDR_paper_cc_and_DDR.pdf.

[CIT0029] Isidore K.K., Aljunid S., Kamigaki T., Hammad K. & Oshirani H., 2012, *Preventing and controlling infectious diseases after Natural disasters*, viewed 04 August 2013, from http://unu.edu/publications/articles/preventing-and-controlling-infectious-diseases-after-natural-disasters.html#info.

[CIT0030] Janossy K., Abas M. & Williams F., 2013, *Climate change impacts on coastal settlements*, viewed 10 December 2013, from http://fluswikien.hfwu.de/index.php/Group_B_-_Collaborative_Climate_Adaption_Project.

[CIT0031] Jonkman S.N., 2005, ‘Global perspectives on loss of human life caused by floods’, *Natural Hazards* 34, 151–175. 10.1007/s11069-004-8891-3

[CIT0032] Kouadio I.K., Aljunid S., Kamigaki T., Hammad K. & Oshitani H., 2012, ‘Infectious diseases following natural disasters: Prevention and control measures’, *Expert Review of Anti-infective Therapy* 10, 95–104. 10.1586/eri.11.155 22149618

[CIT0033] Lau C.L., Smythe L.D., Criag S.B. & Weinstein P., 2010, ‘Climate change, flooding, urbanization and leptospirosis’, *Transactions of Royal Society of Tropical Medicine and Hygiene* 104, 631–638. 10.1016/j.trstmh.2010.07.002 20813388

[CIT0034] Lemos M.C. & Tompkins E.L., 2008, ‘Responding to the risk from climate related disasters’, id21 *highlights Climate Change*. UK: IDS, page 1–4

[CIT0035] LG-STAT, 2012, ‘Abstract of Lagos state statistics’, Lagos State Bureau Of Statistics, M. O. E. P. A. B., Lagos state, Nigeria.

[CIT0036] Li Y., Guo Y. & Yu G., 2013, ‘An analysis of extreme flood events during the past 400 years at Taihu lake, China’, *Journal of Hydrology* 500, 217–225. 10.1016/j.jhydrol.2013.02.028

[CIT0037] Means T., 2018, *The types of flood events and their causes*, viewed 15 August 2018, from https://www.thoughtco.com/the-types-of-flood-events-4059251.

[CIT0038] Monye F.N., 2006, ‘An appraisal of the National Health Insurance Scheme of Nigeria’, *Commonwealth Law Bulletin* 32, 415–427. 10.1080/03050710601074450

[CIT0039] Ndiribe O., 2010, *Federal government establishes 57 disaster reaction units*, Vanguard, viewed 23 August 2018, from https://www.vanguardngr.com/2010/12/fg-establishes-57-disaster-reaction-units/.

[CIT0040] NDMF, 2010, *Nigeria: National Disaster Framework*, Government of Nigeria, Abuja, Nigeria.

[CIT0041] NEMA, 2004a, *Laws of the Federation of Nigeria*, Cap 34, vol 10 Section 6 (1), 1st edn., Government of Nigeria, Abuja.

[CIT0042] NEMA, 2004b, *Laws of the Federation of Nigeria*, Section 4, Cap 34, Government of Nigeria, Abuja.

[CIT0043] NEMA, 2011a, *Disaster risk reduction, National Emergency Management Agency, Lake Nyos Disaster Response Plan*, Government of Nigeria, Abuja.

[CIT0044] NEMA, 2011b, *NEMA, SAR, EEP*, ed. NEMA, Federal Government of Nigeria, Abuja, Nigeria.

[CIT0045] NEMA, n.d.a, *National disaster response plan*, Nigeria, viewed 23 August 2018, from https://www.preventionweb.net/files/21707nigeria.pdf.

[CIT0046] NEMA, n.d.b, National Emergency Management Agency: DRR Profile, viewed on 10 October 2015 from https://www.preventionweb.net/organizations/1176.

[CIT0047] NEMA & UNICEF, 2011, *Planning, research and forecasting. National contingency plan of Nigeria*, Government of Nigeria, Abuja.

[CIT0048] Ngutor K.S., 2015, ‘Implications of recurrent flood episodes in Nigeria on public health: A review’, *Tropical Veterinarian* 31, 1–2.

[CIT0049] Nigeria-Government, 2010, *National disaster framework. Understanding the framework*, viewed 2 February 2015 from https://www.preventionweb.net/english/professional/policies/v.php?id=21708.

[CIT0050] Nigerian Finder, 2013, *The largest city in Africa,* viewed 07 August 2017, from http://nigerianfinder.com/the-largest-city-in-africa/.

[CIT0051] Nkwunonwo U.C., Whitworth M. & Baily B., 2016, ‘A critical review of Lagos urban flood risk management in Lagos region of Nigeria’, *Natural Hazards and Earth System Sciences* 16, 349–369. 10.5194/nhess-16-349-2016

[CIT0052] Nkwunonwo U.C., Whitworth M., Baily B. & Inkpen R., 2014, ‘The development of a simplified model for urban flood risk mitigation in developing countries in: Vulnerability, uncertainty and risk quantification, mitigation and management’, ASCE-ICVRAM-ISUMA conference, Liverpool, July 13–16, 2014, pp. 1116–1127.

[CIT0053] NNHC, 2009, *Nigeria National Health Conference Communique, Abuja Nigeria*, viewed 05 April 2014. From www.ngnhc.org.

[CIT0054] OCHA, 2016, *West Africa: Impacts of the floods*, viewed 19 August 2018, from https://www.humanitarianresponse.info/sites/www.humanitarianresponse.info/files/documents/files/wca_a4_l_impact_of_floods_20160822.pdf.

[CIT0055] Odunuga S., Oyebande L. & Omojola A.S., 2012, ‘Socio-economic indicators and public perceptions on urban flooding in Lagos Nigeria. Hydrology for disaster management’, *Nigerian Association of Hydrological Sciences* 1, 82–96.

[CIT0056] Okoli A.C., 2014, ‘Disaster management and national security in Nigeria: The nexus and the disconnect’, *International Journal of Liberal Arts and Social Science* 2, 21–59.

[CIT0057] Okonkwo I., 2013, *Effective flood plain management in Nigeria: Issues, Benefits and challenges*, viewed 21 March 2014, from http://transparencyng.com/index.php/contributions/60-guest/8548-effective-flood-plain-management-in-nigeria-issues-benefits-and-challenges.

[CIT0058] Oladokun V.O. & Proverbs D., 2016, ‘Flood risk management in Nigeria: A review of the challenges and opportunities’, *International Journal of Safety and Security Engineering* 6, 485–497. 10.2495/SAFE-V6-N3-485-497

[CIT0059] Omoruan A.L., Bamidele A.P. & Phillip O.F., 2009, ‘Social health Insurance and sustainable healthcare reform in Nigeria’, *Ethno Med. 3rd Edition pages 105–10,* 3, 105–110.

[CIT0060] Onwabiko E., 2012, *Human Rights Writers Association of Nigeria*, viewed 12 June 2017, from www.huriwa.Blogspot.com.

[CIT0061] Onwumere O., 2013, *Sanitation and water-borne diseases: Authorities must kick-off practical policies. Available: Sanitation and water-borne diseases: Authorities must kick-off practical policies*, viewed 12 July 2014, from http://www.thenigerianvoice.com/nvnews/124010/1/sanitation-and-water-borne-diseases-authorities-mu.html.

[CIT0062] Opperman J.J., Galloway G.E. & Duvail S., 2013, ‘The multiple benefits of river-floodplain connectivity for people and biodiversity’, in Levin S. (ed.), *Encyclopedia of biodiversity,* 2nd edn., pp. 144–160, Academic Press, Waltham, MA.

[CIT0063] Oshodi L., 2012, *Flood management and governance structure in Lagos, Nigeria*, viewed 22 August 2018, from http://resilient-cities.iclei.org/fileadmin/sites/resilient-cities/files/Resilient_Cities_2013/Oshodi_Paper_Flood_Management_2013_01.pdf.

[CIT0064] Peate I., Wild K. & Nar M., 2013, *Nursing practice: Knowledge and care*, John Wiley & Sons, New Jersey.

[CIT0065] Sessou E., 2012, ‘Flood takes over Lagos, destroys properties’, *Vanguard,* 28 June, viewed 14 June 2014, from https://www.vanguardngr.com/2012/06/flood-takes-over-lagos-road-destroys-properties/.

[CIT0066] Shaba H. A., 2009, *National disaster management system in Nigeria,* viewed 15 January 2014, ochaonline.un.org/OchsLinkClick.aspx?link=ocha&docl.

[CIT0067] Sojobi A., Balogun I. & Salami A., 2015, ‘Climate change in Lagos state, Nigeria: What really changed?’, *Environmental Monitoring and Assessment* 188(10), 1–43. 10.1007/S10661-016-5549-z 27613292

[CIT0068] UN, 2017, *The sustainable development goals report 2017*, United Nations, New York, viewed 19 August 2018, from http://sdgactioncampaign.org/wp-content/uploads/2017/07/TheSustainableDevelopmentGoalsReport2017.pdf.

[CIT0069] UNISDR, 2015, *The human cost of weather related disasters 1995–2015*, United Nations Office for Disaster Risk Reduction, Geneva.

[CIT0070] Welcome M.O., 2011, ‘The Nigerian healthcare system: Need for integrating adequate medical intelligence and surveillance system’, *Journal of Pharm Bioallied Sciences* 3, 470–478. 10.4103/0975-7406.90100 PMC3249694 22219580

[CIT0071] WHO, 2012, *Public health risk assessment and interventions. Flooding disaster: Nigeria*, viewed 20 August 2018, from http://www.who.int/hac/crises/nga/RA_Nigeria_1Nov2012a.pdf.

[CIT0072] WHO, n.d., *Flooding and communicable disease fact sheet*, viewed 19 August 2018, from http://www.who.int/hac/techguidance/ems/flood_cds/en/. 15715138

[CIT0073] Yamin A., 2014, *Why are the poor the most vulnerable to climatic hazards (e.g. floods)? A case study of Pakistan*., Term paper. University of Potsdam, Germany pp. 1–19.

